# The JAK/STAT Pathway and Its Selective Inhibition in the Treatment of Atopic Dermatitis: A Systematic Review

**DOI:** 10.3390/jcm11154431

**Published:** 2022-07-29

**Authors:** Aikaterini Tsiogka, Maria Kyriazopoulou, George Kontochristopoulos, Electra Nicolaidou, Alexander Stratigos, Dimitris Rigopoulos, Stamatios Gregoriou

**Affiliations:** 1st Department of Dermatology-Venereology, Faculty of Medicine, Andreas Sygros Hospital, National and Kapodistrian University of Athens, 16121 Athens, Greece; a.tsiogka@yahoo.com (A.T.); mckyriazopoulou@gmail.com (M.K.); gkontochris@gmail.com (G.K.); electra.nicol@gmail.com (E.N.); alstrat2@gmail.com (A.S.); dimitrisrigopoulos54@gmail.com (D.R.)

**Keywords:** atopic dermatitis, JAK/STAT pathway, treatment, JAK inhibitor, Th2 immune response, Janus kinase, targeted therapy

## Abstract

In recent years, the broadening understanding of the pathogenesis of atopic dermatitis (AD) has led to the development of novel therapeutic molecules, that target core inflammatory components of the disease. The Janus kinase (JAK)/signal transducer and activation of transcription (STAT) pathway constitutes the principal signaling cascade for a large number of cytokines and growth factors and is involved in intracellular signal transduction and subsequent regulation of gene transcription. Current knowledge suggests that the robust activation of the T-helper (Th)-2 [interleukin (IL)-4, IL-5, IL-13, IL-31] and Th22 (IL-22) immune responses in both skin and serum plays a pivotal role in the immunopathogenesis of AD especially at the acute stage, followed by a variable degree of Th1 (interferon-γ, tumor necrosis factor alpha) and Th17 (IL-17) activation in chronic disease. Of note, most of the aforementioned inflammatory cytokines utilize the JAK/STAT pathway for downstream signal transduction, explaining the emerging role of JAK inhibitors in the therapeutic armamentarium of AD. The present systematic review aims to discuss the involvement of JAK/STAT pathway in the pathogenesis of AD and summarize the clinical data available on the efficacy and safety of JAK inhibitors which have been used in the treatment of AD thus far.

## 1. Introduction

Atopic dermatitis (AD) is a chronic inflammatory skin disease with multifactorial pathophysiology, involving strong genetic predisposition, skin barrier dysfunction, microbiome abnormalities, immune dysregulation and extrinsic factors [1]. Current knowledge suggests that robust activation of the T-helper (Th)-2 [interleukin (IL)-4, IL-5, IL-13, IL-31] and Th22 (IL-22) immune responses in both skin and serum play a pivotal role in the immunopathogenesis of AD especially at the acute stage, followed by a variable degree of Th1[interferon-γ (IFN-γ), tumor necrosis factor alpha (TNF-a)] and Th17 (IL-17) activation in chronic disease [1,2]. Moreover, it is known that most of the aforementioned inflammatory cytokines, including the two hallmark Th2 cytokines IL-4 and IL-13, utilize the Janus kinase (JAK)/signal transducer and activation of transcription (STAT) pathway for downstream, intracellular signal transduction [3]. Apart from immune regulation, the JAK/STAT pathway is involved in multiple biological processes, such as hematopoiesis, cell proliferation, differentiation and apoptosis [4].

The JAK family comprises four different cytoplasmatic tyrosine kinases, the JAK1, JAK2, JAK3 and tyrosine kinase-2 (TYK2), which are linked to the intracellular domains of multiple transmembrane cytokine receptors. Their function is coupled to the STAT family, which comprises seven intracellular transcription factors, the STAT1, STAT2, STAT3, STAT4, STAT5a, STAT5b, and STAT6. These proteins have an N-terminal domain, a DNA-binding domain, and a C-terminal transactivation domain, and contribute to cellular immunity, proliferation, apoptosis and differentiation [5]. Binding of the signaling cytokines to their receptors leads to the activation of the associated JAKs and subsequent phosphorylation of STATs, which, in turn, separate from the receptor, dimerize and translocate to the cell nucleus to induce gene transcription after binding to specific DNA sequences [5]. Of note, more than 50 cytokines signals via the JAK/STAT pathway have been identified, since each JAK may be associated with multiple types of cytokine receptors and, thus, exhibit a different downstream effect on cytokine signaling. Similarly, members of the STAT family may interact with different JAKs, with STAT1 and STAT4 being involved in Th1 and STAT3 and STAT6 in Th2 and Th17 immune responses, respectively [6,7,8].

The broadening understanding of AD pathogenesis in recent years has led to the development of novel therapeutic molecules, that target core inflammatory components of the disease, including the JAK inhibitors. The present review aims to discuss the involvement of the JAK/STAT pathway in the pathogenesis of AD as well as the emerging role of JAK inhibitors in the therapeutic armamentarium of AD. 

## 2. Materials and Methods

**Scheme 1:**
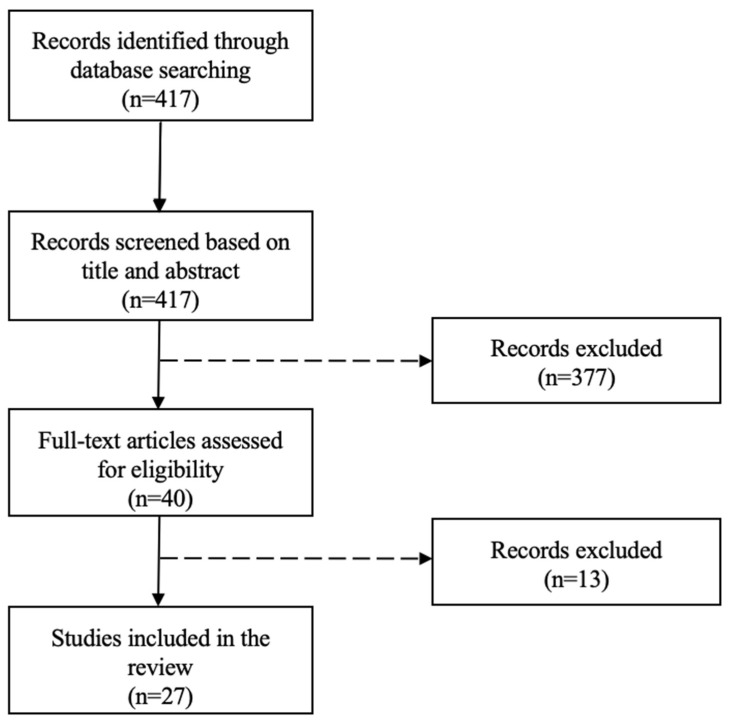
Flow diagram.

The MEDLINE electronic database was searched systematically via PubMed for randomized controlled trials (RCTs) assessing the efficacy and/or safety of JAK inhibitors in AD from inception to May 2022. The search strategy is summarized in Table 1. Non-human studies and articles reporting subgroup and post hoc analyses of data extracted from the pivotal studies were excluded. The initial search yielded 417 articles (no filters used). After title and abstract review (conducted by two independent reviewers, A.T., M.K), 40 articles were selected for full text review. Discrepancies between the two authors that performed the quality assessment of the included studies, were solved by the contribution of a third author (S.G.). Finally, 27 studies were included in the systematic review after the exclusion of irrelevant studies (Table 2, Scheme 1). Reference lists of the included articles as well as the database of clinical trials of the US national library of medicine (https://www.clinicaltrials.gov, accessed on 10 June 2022) were also screened in order to detect any additional RCTs (Table 2). The following data were extracted: JAK inhibitor used, study population, treatment period, disease assessment scores and safety reports.

This systematic review was performed according to the Preferred Reporting Items for Systematic reviews and Meta-Analyses (PRISMA). Apart from the systematic review on the efficacy and safety of JAK inhibitors in AD, a narrative review regarding the involvement of the JAK/STAT pathway in AD was performed and is addressed below.

## 3. The JAK/STAT Pathway in AD

### 3.1. Overregulation of Th2 and Th22 Immune Responses

The involvement of Th2 immune response in AD pathogenesis is well established and is associated with increased signaling through all four JAKs. Mouse models support that IL-4 is required for the production of Th2-related cytokines, including IL-4, IL-5, IL-13 and IL-31, which play a critical role in the development of AD lesions [36]. There are two types of IL-4 receptors; the type 1 receptor is comprised of an IL-4Rα and a γ chain and the type 2 receptor of an IL-4Rα and an IL-13Ra1 chain. The latter functions also as an IL-13 receptor. Cytokine binding to type 1 receptors leads to the phosphorylation of JAK1 and JAK3, resulting in activation of STAT6, while binding to type 2 receptors induces JAK1 and TYK2 expression and subsequent activation of STAT6 and STAT3 [36]. The STAT6 protein has been found to regulate T-cell proliferation and Th2 differentiation through the upregulated expression of the Th2-specific transcription factor GATA3, but also the promotion of immunoglobulin (Ig) class switching to IgE and IgG1 in B cells [37]. For that reason, various STAT6 polymorphisms have been related to high levels of IgE and greater susceptibility to AD [38]. The Interleukin-5-mediated pathway utilizes JAK1 and JAK2 as well as STAT1, STAT2 and STAT5 to transduce its signal [39,40,41]. Finally, the intracellular signaling of IL-31, as well as of thymic stromal lymphopoietin (TSLP), a highly pruritogenic protein, which also promotes the Th2 differentiation, is transmitted by JAK1 and JAK2, with subsequent involvement of STAT1, STAT3 and STAT5 [42,43].

The JAK/STAT-dependent IL-4 and IL-13 signaling is also very important in the dysregulation of keratinocyte function in AD, due to the downregulation of several barrier proteins [42]. For example, IL-4 and IL-13 downregulate loricrin and involucrin in keratinocytes, while they have been also found to reduce the expression of filaggrin in patients who did not carry the known filaggrin mutations [44]. Involucrin and loricrin are structural proteins that contribute to the protective barrier function of the stratum corneum. The superficial spinous cells express involucrin which cross-links to form the cornified envelope scaffolding. Loricrin in turn is cross-linked to involucrin and is expressed in the superficial granular cells forming composite keratohyalin granules or L granules along with profilaggrin [45].

Moreover, downstream signaling of IL-4 and IL-13 modulates the expression of innate immune response genes, including cathelicidin and β-defensins, enhancing the susceptibility to skin infections, for example with staphylococcus aureus, which, in turn, may exacerbate AD by acting on keratinocytes and immune cells [46,47,48].

IL-4 and IL-13 reduce the expression of filaggrin, loricrin, involucrin and lipid components of the skin barrier [45]. This barrier dysfunction contributes to *Staphylococcus aureus* colonization and subsequent inflammation. In addition, activation of the IL-13/IL-4-JAK-STAT6/STAT3 axis inhibits the aryl-hydrocarbon receptor mediated up-regulation of filaggrin, loricrin, and involucrin and also inhibits the cytoplasmic-to-nuclear translocation of the transcription factor Ovo-like-1 (OVOL1) that promotes the expression of filaggrin and loricrin [49]. Moreover, it induces keratinocytes to produce periostin that subsequently upregulates the IL-24 expression and IL-24 inhibits the expression of filaggrin via STAT3 activation [49]. The Th22 immune response is also implicated in AD pathogenesis and is particularly increased in chronic AD lesions [2]. IL-22 promotes skin modification and it has been identified as a key mediator of epidermal hyperplasia. The binding of IL-22 to its receptor leads to the phosphorylation of JAK1 and TYK2, activating STAT1/STAT3/STAT5 [50,51].

**Figure 1:**
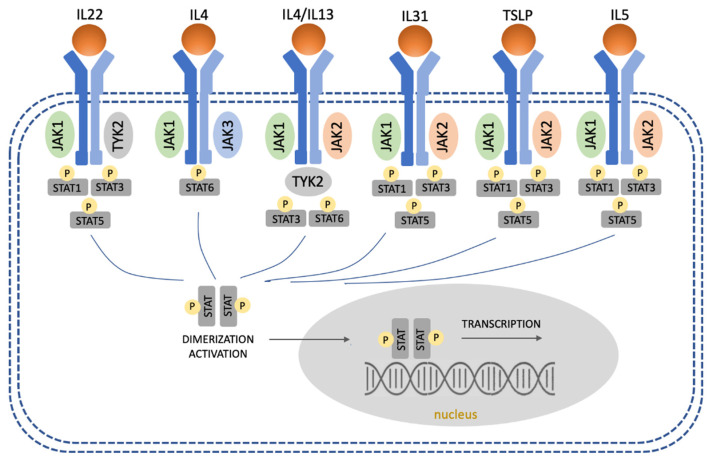
The JAK/STAT signaling pathway in atopic dermatitis. Cytokine binding to each receptor triggers the activation of respective JAKs, leading to phosphorylation of STATs and subsequent dimerization, translocation into the nucleus and regulation of gene transcription. IL: interleukin; JAK: janus kinase; STAT: signal transducer and activation of transcription.

Figure 1 illustrates the involvement of the JAK/STAT signaling pathway in AD.

### 3.2. Regulation of Th1 and Th17 Immune Responses

Although the main cytokines that are involved in AD pathogenesis are the Th2 cytokines, Th1 and Th17 immune responses likely play a pathogenetic role in certain AD subtypes and in chronic AD lesions [52]. The hallmark cytokine of Th1 is IFN-γ, a protein that utilizes STAT1 for signal transduction. Moreover, STAT4 is necessary for IL-12 signaling, which directs the differentiation of naïve T helper cells into Th1 cells [53,54].

Th17 cytokines are elevated in blood and lesional AD and may exaggerate skin both acute and chronic AD [55]. After binding of IL17 to its receptor, the adaptor protein Act1 interacts with the TNF-receptor-associated factor 6 and the transforming growth factor-β-activated kinase 1, leading to activation of nuclear factor (NF)-kB and p38/mitogen-activated protein kinases. This implies that IL17-mediated inflammation does not depend on JAK/STAT signaling [50]. However, literature data suggest that STAT3 is necessary for the induction of retinoic acid receptor-related orphan receptor γ (RORγt), a Th17-specific transcription factor that induces IL17 expression [53].

### 3.3. Suppression of Regulatory T Cells

Regulatory T cells (Tregs) in the skin are known to regulate the immune response in order to maintain immune tolerance to self-antigens and prevent excessive responses to foreign antigens. In AD, they play an important role in controlling skin inflammation and its expansion to the lungs according to a murine AD model, where the depletion of Tregs has led to disease exacerbation, due to Th2 expansion and IgE elevation [56]. Differentiation, proliferation and maintenance of Tregs depend on the transcription factor Foxp3 and mutations leading to altered expression or function of Foxp3 may lead to a severe autoimmune disease called immunodysregulation polyendocrinopathy enteropathy X-linked syndrome [56]. In a mouse model, Foxp3 has been found to have a short half-life and to be controlled by the JAK/STAT signaling [57]. Foxp3 has been found to be suppressed by GATA3, the main regulator of Th2 cells, which is upregulated by STAT6, as mentioned above. In addition, STAT5A/B and STAT3 may also be involved in the downregulation of Foxp3, all explaining why Treg function is suppressed in AD [42,58].

### 3.4. Stimulation of Eosinophils and Mast Cells

Interleukin-31 has been found to delay the apoptosis of eosinophils and significantly induce the secretion of pro-inflammatory cytokines and chemokines after binding on the IL-31 receptor A and subsequent activation of STAT3 in eosinophils [59]. Moreover, upregulation of IL-5 has been shown to induce eosinophilia and regulate the proliferation, survival and functions of eosinophils in a JAK2-STAT1/STAT5-dependent manner [41,60]. Interleukin-9 has been found to be increased in the lesional skin of AD patients and it has been associated with localized stimulation of VEGF from mast cells through activation of STAT3 in vitro [51,61]. Finally, the JAK/STAT pathway is involved in the maturation of B cells and their differentiation into plasma cells with the subsequent secretion of IgE, which activates cutaneous mast cells to release histamine [6,62].

Apart from the IgE-dependent mast cell activation, the JAK/STAT pathway is also involved in mast cell homeostasis and proliferation. The binding of the stem cell factor to c-kit on the human mast cell surface leads to JAK2 activation and subsequent STAT5 and STAT6 phosphorylation, which are all involved in mast cell development, survival and proliferation [63]. In addition, the same molecules (JAK2/STAT5/STAT6) may be activated by IL3 as well, a cytokine that regulates the mast cell response against antigens. Besides, the important role of the JAK/STAT pathway in mast cells is underlined by the consequences of the mutations of these molecules, including those that are found in mastocytosis [63].

## 4. JAK Inhibitors for the Treatment of AD

JAK inhibitors are small molecules, which are classified into first generation and newer JAK inhibitors. First generation JAK inhibitors bind to all three JAKs, whereas newer JAK inhibitors are more selective and demonstrate activity against specific JAKs [52]. JAK inhibitors can be administered orally due to their size and chemical structure. Some of them, including baricitinib and upadacitinib, are eliminated primarily by renal excretion and are characterized by a half-life between 9 and 14 h, making a single daily administration sufficient. On the other hand, JAK inhibitors, such as ruxolitinib and tofacitinib are metabolized in the liver and have a shorter half-life (approximately 3 h). Therefore, they should be administered twice daily [64]. All registered clinical trials on topical and systemic JAK inhibitors are listed in Table 2.

## 5. Topical JAK Inhibitors

To date, a number of trials assessed the efficacy and safety of several topical formulations in AD. Delgocitinib is a first-generation topical pan-JAK inhibitor, which was the first JAK inhibitor approved for AD in adults (0.5% ointment) and children (0.5% and 0.25% ointment) in Japan [65]. The two phase II clinical trials conducted in Japan showed the efficacy of delgocitinib in both adults and children (2–15 years of age) with a favorable safety profile [9,10]. In a double-blind, phase III RCT (*n* = 158, patients over 16 years of age with moderate-to-severe AD), delgocitinib 0.5% ointment met the primary endpoint, achieving improvement of percentage change from baseline modified EASI (%mEASI) at week 4 (44.3% vs. 1.7% for delgocitinib 0.5% vs. vehicle, respectively) [11]. Moreover, 95% of patients achieved at least 50% in the mEASI (mEASI-50) and 49% at least 75% in the mEASI (mEASI-75) during the 24-week extension phase. Regarding safety, the overall rate of treatment-related AEs in the delgocitinib group was 15.4%, with only eczema herpeticum occurring in three patients [11]. Similar positive results with a good safety profile were supported by a further phase III RCT with subsequent long-term extension (56 weeks) in pediatric patients between 2–15 years of age with AD [12].

Tofacitinib is another small molecule that inhibits selectively JAK1 and JAK3. In a double-blind phase IIa RCT evaluating tofacitinib 2% ointment in 69 adult patients with mild-to-moderate AD, there was an 81.7% improvement in Eczema Area and Severity Index (EASI), compared to 29.9% with the vehicle, as assessed at week 4. Tofacitinib also met several secondary endpoints, including improvement in Investigator’s Global Assessment (IGA) score, Body Surface Area (BSA) and itch [13].

Ruxolitinib, a first-generation inhibitor of JAK1 and JAK2, was the first approved oral JAK inhibitor for the treatment of hematologic disorders, while it has also been investigated for the treatment of AD in the form of topical formulation. Following the positive results of a dose-ranging, double-blind, phase IIb RCT, the efficacy of ruxolitinib was evaluated in two parallel phase III trials [TRuE AD1 (*n* = 631), TRuE AD2 (*n* = 618)], where ruxolitinib 1.5% vs. 0.75% cream vs. vehicle were administered for eight weeks in adolescents and adults ≥ 12 years of age with mild-to-moderate AD [14]. Overall, the primary endpoint of IGA 0/1 with ≥2-grade improvement from baseline at week 8 was met by almost half of the patients in the active-agent groups. Moreover, both formulations met the secondary endpoints of EASI-75 and the critically relevant reduction of itch and exhibited rates of treatment-related AEs comparable to the vehicle (26.3% vs. 29.4% vs. 33.6%), with no serious AEs reported [14].

Other topical JAC inhibitors, which are under investigation for the treatment of AD comprise Cerdulatinib (DMVT-502, dual JAK and spleen tyrosine kinase inhibitor), Brepocitinib (JAK1 and TYK2 inhibitor), ATI-502 (JAK1/3 inhibitor) and SHR0302 (JAK1 inhibitor) [66,67,68,69].

## 6. Systemic JAK Inhibitors

### 6.1. Baricitinib

Baricitinib is a selective JAK1 and JAK2 inhibitor, with less affinity for JAK3 and TYK2. The recommended dosage is 4 mg once daily but it may be reduced to 2 mg daily if the disease is well controlled in patients over 75 years of age, and in cases of renal impairment or risk of infections [70]. The results of a phase II, placebo-controlled RCT (*n* = 124) highlighted the efficacy of baricitinib combined with topical corticosteroids (TCS) in adults with moderate to severe AD, based on the observed achievement of EASI-50 at week 16 [15].

Subsequently, the efficacy and safety of baricitinib have been evaluated in two parallel, independent, multicentric, double-blinded, phase III RCTs [BREEZE-AD1 (*n* = 624) and BREEZE-AD2 (*n* = 615)] [16]. In both studies, adult patients with moderate to severe AD were randomized 2:1:1:1 to receive baricitinib 1 mg, 2 mg, 4 mg or placebo once daily for 16 weeks. In BREEZE-AD1, the primary endpoint of IGA 0/1 at week 16 was met by more patients on baricitinib arms compared with placebo [16.8% in baricitinib 4 mg, 11.4% in baricitinib 2 mg, 11.8% in baricitinib 1 mg, 4.8% in the placebo group, respectively]. Similarly, the same primary endpoint was met by 13.8% in baricitinib 4 mg, 10.6% in baricitinib 2 mg, 8.8% in baricitinib 1 mg, and 4.5% in the placebo group, respectively, in the BREEZE-AD2 study [16]. Moreover, baricitinib 4 mg achieved consistently all secondary endpoints in both trials, including EASI-75, improvement of skin pain and night awakenings and rapid relief of pruritus within the first week of therapy, suggesting the superiority of this dosing regimen compared to the others or placebo. After week 16, responders or partial responders from the baricitinib 4 mg and 2 mg groups (IGA ≤ 2) were further evaluated in a long-term extension study (BREEZE-AD3) and showed further improvement with a similar safety profile up to week 68, suggesting a sustained long-term benefit for these patients [17]. The efficacy of baricitinib monotherapy in adults with moderate-to-severe AD was evaluated in a further North American RCT [BREEZE-AD5 (*n* = 440)], showing similar efficacy and safety findings to those reported in the previous baricitinib AD studies [19].

Two multicenter, double-blinded, placebo-controlled phase III RCTs [BREEZE-AD4 (*n* = 463), BREEZE-AD7 (*n* = 329)] assessed the efficacy of baricitinib (4 mg and 2 mg) vs. placebo combined with TCS in adult patients with moderate to severe AD [18,20]. A statistically significant improvement in AD at week 16 was observed only in the baricitinib 4 mg group in both studies based on the endpoint of EASI-75 in BREEZE-AD4 (baricitinib 4 mg vs. 2 mg vs. placebo; 31.5% vs. 27.6% vs. 17.2%, respectively) and IGA 0/1 in BREEZE-AD7 (baricitinib 4 mg vs. 2 mg vs. placebo; 31% vs. 24% vs. 15%, respectively). Improvements could be maintained through 52 weeks of treatment [18,20].

Regarding the safety profile of baricitinib, treatment-related AEs were observed in 54% to 58% of patients, they were mild to moderate in severity and were not found to differ significantly between the four study arms in the two pivotal studies BREEZE-AD1 and BREEZE-AD2 [16]. The most frequently reported AEs were nasopharyngitis, headache and asymptomatic increased creatine phosphokinase (CPK) levels. Herpes simplex infection was observed in 7.2% vs. 3.3% vs. 5.5% vs. 1.2% of patients receiving baricitinib 4 mg vs. 2 mg vs. 1 mg vs. placebo in the BREEZE-AD1 study, but it was not so frequent in the BREEZE-AD2 study. Overall, no opportunistic infections, malignancies, thromboembolic events and major adverse cardiovascular events were reported in any study arm in both studies [16]. Pooled safety data of baricitinib were reported in a meta-analysis of six RCTs and two long-term extension studies (total *n* = 2531) highlighting a good overall safety profile [71]. The most common AEs were found to be upper respiratory tract and herpes simplex virus infections, exhibiting higher frequency in the baricitinib 4 mg group, while the most common serious AEs were eczema herpeticum [*n* = 11, adjusted incidence rate (IR) per 100 patient-years: 0.5], cellulitis (*n* = 6, IR: 0.3) and pneumonia (*n* = 3, IR: 0.1). Overall, two major adverse cardiovascular events and three thromboembolic events were identified in the total baricitinib data set [71].

### 6.2. Upadacitinib

Upadacitinib is a selective JAK1-inhibitor, which has been approved for the treatment of moderate to severe AD in adults and adolescents (≥12 years). The recommended dosage is 15–30 mg once daily based on disease severity, while a dosage of 15 mg once daily should be also recommended in patients ≤18 or ≥65 years of age [72]. In a multicenter, double-blinded, placebo-controlled phase IIb RCT (*n* = 164) patients were treated with varying doses of upadacitinib (7.5 mg, 15 mg, 30 mg) for 16 weeks [21]. The improvement of the EASI score was dose-dependent, while equal and rapid improvement of pruritus was observed in all active-treatment groups [21].

Two parallel, multicentric, double-blinded phase III RCTs [Measure Up 1 (*n* = 847), Measure Up 2 (*n* = 836)] evaluated the efficacy and safety of upadacitinib in moderate to severe AD in adults and adolescents (12–75 years) [22]. In both studies, patients were randomly assigned to receive either upadacitinib 30 mg or 15 mg or placebo once daily for 16 weeks. All upadacitinib groups met the primary endpoints of EASI-75 and IGA 0/1 at week 16. In particular, in Measure Up 1, EASI-75 was achieved by 80%. 70% and 16%, while IGA 0/1 by 48%, 62% and 8% of patients receiving upadacitinib 30 mg, 15 mg and placebo, respectively. In Measure Up 2, EASI-75 was achieved by 73%. 60% and 13%, while IGA 0/1 by 39%, 52% and 5% of patients receiving upadacitinib 30 mg, 15 mg and placebo, respectively. In addition, the secondary endpoints were also met in all active-treatment groups in both studies, including the achievement of EASI-90 and reduction of itch [22]. The analysis of the follow-up data of these two RCTs demonstrated sustained efficacy and a favorable safety profile of upadacitinib through week 52 [23].

The efficacy of upadacitinib combined with TCS in patients aged 12 to 75 years old suffering from moderate to severe AD has been assessed in a further double-blinded, placebo-controlled, phase III RCT [AD Up (*n* = 901)]. Eligible patients were randomized 1:1:1 to receive TCS combined with either upadacitinib 30 mg, or upadacitinib 15 mg or placebo for 52 weeks [24,25]. Both upadacitinib groups met the two primary endpoints of EASI-75 and IGA 0/1 at week 16. EASI-75 was achieved by 77.1%, 64.6% and 26.4% of patients receiving upadacitinib 30 mg, 15 mg and placebo, respectively, (*p* < 0.0001) while IGA 0/1 by 58.6%, 39.6% and 10.9% of those receiving upadacitinib 30 mg, 15 mg and placebo, respectively, (*p* < 0.001) [24,25]. Moreover, both upadacitinib arms demonstrated a rapid onset of action as soon as week 2 regarding skin clearance and week 1 regarding pruritus, which could be maintained through week 52 [24].

Upadacitinib has been also tested in a head-to-head phase III RCT, that compared upadacitinib 30 mg once daily with dupilumab 300 mg every 2 weeks for 24 weeks in adults with moderate-to-severe AD [Heads Up (*n* = 692)] [26]. More patients from the upadacitinib group achieved the primary endpoint of EASI-75 at week 16 compared to dupilumab [71% and 61.1%, respectively (*p* = 0.006)]. In addition, the secondary endpoints (EASI-75 at week 2, EASI-100 at week 16, improvement of pruritus) demonstrated the superiority of upadacitinib vs. dupilumab [26].

Regarding safety, the most commonly reported treatment-related AEs in the phase III trials were acne, nasopharyngitis, headache, upper respiratory tract infections and CPK elevation. There were isolated herpetic infections in both Measure Up 1 and Measure 2 trials (Measure Up 1: herpes zoster in six patients of the 30 mg group and five of the 15 mg group; eczema herpeticum in three cases of the 30 mg arm and four of the placebo arm; Measure Up 2: herpes zoster in three patients of the 30 mg arm, in six patients of the 15 mg arm and two patients of placebo arm; eczema herpeticum in three cases of the 15 mg arm). Apart from one venous thromboembolism in Measure Up 2, there were no opportunistic infections, active tuberculosis, or deaths [22]. The AE profile of upadacitinib was similar also in the phase III, AD up trial, in which there was no difference in AEs incidence between the treatment and placebo arms [24,25]. In the Heads Up study, the rates of herpes zoster, eczema herpeticum, other serious infections and laboratory abnormalities were higher in the upadacitinib arm and consistent with the aforementioned phase III studies, whereas rates of conjunctivitis and injection-site reactions predominated in the dupilumab arm [26]. Finally, a double-blinded, placebo-controlled phase III RCT, designed to evaluate the safety of upadacitinib combined with TCS in Japanese adult patients with moderate to severe AD [Rising up (*n* = 272)], demonstrated consistent results with those of prior multicentric phase II and III studies, as reported above [27].

### 6.3. Abrocitinib

Abrocitinib is a selective JAK1 inhibitor, that has already been evaluated in phase III RCTs in adolescents and adults. A dose-ranging, double-blinded, placebo-controlled phase IIb RCT (*n* = 267) evaluated the efficacy of abrocitinib 200 mg vs. 100 mg vs. 30 mg vs. 10 mg vs. placebo once daily for 12 weeks in adults with moderate to severe AD [28]. The primary endpoint of IGA0/1 was achieved by 43.8% vs. 29.6% vs. 5.8% of patients from the abrocitinib 200 mg vs. 100 mg vs. placebo, respectively. Reduction in the EASI score (secondary endpoint) was also dose-dependent, with greater reductions being observed in patients treated with abrocitinib 200 mg or 100 mg [28].

Two parallel, multicenter, double-blinded, placebo-controlled phase III RCTs evaluated the efficacy and safety of abrocitinib 200 mg vs. 100 mg vs. placebo once daily for 12 weeks in patients ≥ 12 years of age with moderate-to-severe AD [JADE MONO-1 (*n* = 387), JADE MONO-2 (*n* = 391)] [29,30]. Active-treatment groups in both studies met the primary endpoints as early as week 2 as well as the secondary endpoints, including rapid improvement of pruritus. In particular, EASI-75 was achieved by 63%, 40%, 12% and IGA 0/1 by 44%, 24%, 8% of patients receiving abrocitinib 200 mg, 100 mg and placebo, respectively, in JADE MONO-1 trial [29]. Similarly, EASI-75 was achieved by 61%, 45%, 10% and IGA 0/1 by 38%, 28%, 9% of patients receiving abrocitinib 200 mg, 100 mg and placebo, respectively, in the JADE MONO-2 trial [30]. Another double-blinded, placebo-controlled phase III RCT compared abrocitinib 200 mg vs. 100 mg vs. placebo with additional use of topical therapy for 12 weeks in adolescents between 12 and 17 years of age [JADE TEEN (*n* = 285)] [31]. The primary endpoints were met by substantially more patients from the abrocitinib groups (EASI-75: 72% vs. 68.5% vs. 41.5%; IGA 0/1: 46.2% vs. 41.6% vs. 24.5%, for abrocitinib 200 mg vs. 100 mg vs. placebo, respectively) [31]. In order to assess the effect of treatment modification on AD symptoms, a phase III RCT [JADE REGIMEN (*n* = 798)] evaluated the response of continuous abrocitinib treatment vs. dose reduction or withdrawal and the effect of treatment recommencement following flare in patients who responded previously to monotherapy with abrocitinib 200 mg. All of the aforementioned therapeutic regimens were proven to be effective and most responders continuing abrocitinib did not flare [32].

A following placebo-controlled phase III RCT evaluated the efficacy of abrocitinib compared to dupilumab in adult patients with moderate to severe AD [JADE COMPARE (*n* = 838)] [33]. Study participants received either abrocitinib 200 mg daily or 100 mg daily or dupilumab 300 mg every other week or a placebo for 16 weeks, while the concomitant use of topical therapy was not prohibited, unlike JADE MONO studies. The achievement rate of primary endpoints (EASI-75 and IGA0/1) was found to be statistically significant only between abrocitinib vs placebo [EASI-75: 70.3% (*p* < 0.001) vs. 58.7% (*p* < 0.001) vs. 58.1% (*p* > 0.05) vs. 27.1%; IGA 0/1: 48.4% (*p* < 0.001) vs. 36.6% (*p* < 0.001) vs. 36.5% vs. 14%; for abrocitinib 200 mg vs. 100 mg vs. dupilumab vs. placebo, respectively]. Statistical comparison between abrocitinib and dupilumab, regarding IGA0/1 and EASI-75 was not among the trial endpoints and was not assessed. Abrocitinib 200 mg was also found to be superior to dupilumab with respect to itch response at week 2 but both treatments were equal as regards most other secondary endpoints at week 16 [33]. Abrocitinib has been also found to be effective in patients with moderate to severe AD, who previously received dupilumab, according to the results of the JADE EXTEND (*n* = 203) clinical trial [34].

Safety analysis revealed a greater number of treatment-related AEs in abrocitinib groups in both JADE MONO trials (JADE MONO-1: 69–78% vs. 57%; JADE MONO-2: 63–66% vs. 42%; for abrocitinib groups vs. placebo, respectively), while similar percentages of AEs occurred also in the JADE COMPARE trial [29,30,33]. However, the rate of SAEs was similar between all groups including placebo (between 3.2% and 3.9% in JADE MONO-1 and 1.3% and 3.2% in JADE MONO-2). There was only one sudden cardiac death in the 100 mg abrocitinib group of the JADE MONO-2 trial during the follow-up period, which was not attributed to study treatment. In both studies, nasopharyngitis, nausea, headache and upper respiratory tract infections were the most commonly documented AEs. Herpes simplex infection was only observed in the abrocitinib groups in the JADE MONO trials and in the abrocitinib as well as placebo group in the JADE COMPARE trial. Thrombocythemia was reported in both trials, similarly to the previous phase II study, but it was self-limiting despite treatment continuation [29,30,33].

### 6.4. Other Oral JAKs

The efficacy and safety of SHR0302, a highly selective JAK1 inhibitor, in adult atopic dermatitis has been assessed in a double-blinded, placebo-controlled phase II RCT (*n* = 105) conducted in China between October 2019 and August 2020. Patients were randomized 1:1:1 to receive SHR0302 8 mg vs. 4 mg vs. placebo for 12 weeks. The primary endpoint of IGA 0/1 was met by 54.3% (*p* < 0.001) vs. 25.7% (*p* = 0.022) vs. 5.7% of patients in the 8 mg vs. 4 mg vs. placebo group, respectively. Regarding the safety profile, no serious AEs or infections were reported, and most AEs were mild in severity [35].

## 7. Comparative Data Regarding the Efficacy and Safety of JAK Inhibitors in AD

Despite the growing utilization of JAK inhibitors in AD based on the availability of efficacy and safety data from phase III RCTs, head-to-head comparisons of different JAK inhibitors are lacking and comparative data may yet only be derived from published meta-analyses.

A network meta-analysis of six RCTs conducted by Zhang et al. in 2021 compared the efficacy of six JAK inhibitors (abrocitinib, baricitinib, upadacitinib, tofacitinib, ruxolitinib, delgocitinib) based on the achievement of EASI-50. Among all included, oral upadacitinib 30 mg twice daily exhibited the best efficacy, while topical delgocitinib 3% twice daily was found to be superior to other topical agents [73]. A similar meta-analysis of 14 RCTs conducted by Li et al. in 2021 evaluated the efficacy of topical and oral JAK inhibitors based on the weighted mean difference (WMD) of EASI and IGA response [74]. The largest improvement of both markers was achieved at week 4 and topical agents were found to be significantly more efficacious than systemic ones. Upadacitinib was the most effective in reducing EASI while abrocitinib was most effective regarding IGA response at week 4 of treatment. Although abrocitinib showed a higher incidence of AEs, the relative risk of AEs leading to treatment discontinuation did not differ among all JAK inhibitors [74]. Finally, Silverberg et al. conducted a network meta-analysis of 11 RCTs to assess the efficacy of available targeted systemic therapies (abrocitinib, upadacitinib, baricitinib, dupilumab, tralokinumab) without topical therapy on moderate to severe AD. The study included analysis of IGA0/1, EASI-75, EASI-90 and assessment of pruritus at weeks 12 and 16 and at earlier time points. In all, upadacitinib 30 mg daily was found to be the most efficacious systemic treatment (based on all endpoints), followed by upadacitinib 15 mg daily and abrocitinib 200 mg daily [75].

In September 2021, the US Food and Drug Administration (FDA) reported that there is an increased risk of serious heart-related events, including stroke, myocardial infarction, blood clots and death with oral tofacitinib, based on the results of the ORAL surveillance study of tofacitinib versus TNFa in patients with rheumatoid arthritis. Recently, the “black box warning” was added to all currently approved oral JAK inhibitors, including baricitinib and upadacitinib, based on the fact that these agents share common pharmacological mechanisms with tofacitinib [76]. However, larger safety clinical trials must elucidate if this extrapolation of data from the pan-JAK inhibitor tofacitinib to the selective JAK inhibitors is justified, or if the differences in their specificity for the four JAKs as well as in the pathophysiology of the treated disease (Table 3) is related to different safety profiles among these agents [72,77,78].

## 8. Conclusions

Baricitinib, Upadacitinib and Abrocitinib have been approved for the treatment of moderate/severe atopic dermatitis in patient candidates for systemic therapy by both the Food and Drug Administration and the European Medicines Agency. Selective targeting of the JAK/STAT pathway allows for the control of multiple cytokines involved in AD pathogenesis, such as IL4, 13, 31, 22 and TSLP rather than blocking a single inflammatory interleukin, such as with monoclonal antibodies. Furthermore, the broad-spectrum immunosuppression caused by cyclosporin, azathioprine and mycophenolate is avoided. Literature suggests that this mode of action along with the pharmacokinetics of JAK inhibitors might account for excellent efficacy, rapid onset and long-term control of eczematous lesions and itch in AD combined with an acceptable safety profile, despite the need for screening and monitoring. Data are becoming available for both the JAK/STAT pathway and the IL blocking, along with the investigation of the other pathways involved in AD pathogenesis, such as TSLP and OX40 blockade, resulting in novel insights into the immunological components of the disorder, as well as a new era of treatment options for the patients.

## Figures and Tables

**Table 1:** MEDLINE search strategy *.

Search Terms	Results
JAK inhibitor	9898
JAK/STAT pathway	8894
Tofacitinib	2314
Delgocitinib	46
Ruxolitinib	2240
Baricitinib	885
Upadacitinib	340
Abrocitinib	88
JAK inhibitor OR JAK/STAT pathway OR tofacitinib OR delgocitinib OR ruxolitinib OR baricitinib OR upadacitinib OR abrocitinib	18,427
Atopic dermatitis	32,379
Atopic dermatitis AND (JAK inhibitor OR JAK/STAT pathway OR tofacitinib OR delgocitinib OR ruxolitinib OR baricitinib OR upadacitinib OR abrocitinib)	417

JAK: Janus kinase, STAT: signal transducer and activation of transcription. * via PubMed, performed on 31 May 2022.

**Table 2:** Selected studies and registered clinical trials on currently available JAK inhibitors for the treatment of atopic dermatitis.

Study Identifier	Phase	Study Group	Intervention	Primary Endpoint(s)
Delgocitinib (topical pan-JAK inhibitor)				
JapicCTI-173553 [9]	II	2–15	Delgocitinib (oint), vehicle	mEASI change at week 4: - 0.25% oint: −54.2% - 0.5% oint: −61.8%
JapicCTI-152887 [10]	II	16–65	Delgocitinib (oint), tacrolimus, vehicle	mEASI change at week 4: - 0.25% oint: −41.7% - 0.5% oint: −57.1% - 1% oint: 54.9% - 3% oint: 72.9% - vehicle: −12.2%
JapicCTI-173554 [11]	III	≥16	Delgocitinib (oint), vehicle	mEASI change at week 4 (part 1): - 0.5% oint: −44.3% - vehicle: −1.7% mEASI improvement maintained through the 24-week open-label extension period (part 2)
JapicCTI-184064 [12]	III	2–15	Delgocitinib (oint), vehicle	mEASI change at week 4 (part 1): - 0.25% oint: −39.3% - vehicle: −10.9% mEASI improvement maintained through the 56-week open-label extension period (part 2)
Tofacitinib (topical pan-JAK inhibitor)				
NCT02001181 [13]	II	18–60	Tofacitinib (oint), vehicle	EASI change at week 4: - 2% oint: −81.7% - vehicle: −29.9%
Ruxolitinib (topical selective JAK1/2 inhibitor)				
NCT03745638 (TRuE AD1) [14]	III	≥12	Ruxolitinib (cream), vehicle	Patients with IGA 1/0 at week 8: - 0.75% cream: 50% - 1.5% cream: 53.8% - vehicle: 15.1%
NCT03745651 (TRuE AD2) [14]	III	≥12	Ruxolitinib (cream), vehicle	Patients with IGA 1/0 at week 8: - 0.75% cream: 39% - 1.5% cream: 51.3% - vehicle: 7.6%
Baricitinib (systemic selective JAK1/2 inhibitor)				
NCT02576938 [15]	II	≥18 years	Baricitinib, placebo, TCS	Patients with EASI-50 at week 16: - 2 mg: 57% - 4 mg: 61% - placebo: 37%
NCT03334396 (BREEZE-AD1) [16]	III	≥18 years	Baricitinib, placebo	Patients with IGA 0/1 at week 16: - 1 mg: 11.8% - 2 mg: 11.4% - 4 mg: 16.8% - placebo: 4.8%
NCT03334422 (BREEZE-AD2) [16]	III	≥18 years	Baricitinib, placebo	Patients with IGA 0/1 at week 16: - 1 mg: 8.8% - 2 mg: 10.6% - 4 mg: 13.8% - placebo: 4.5%
NCT03334435 (BREEZE-AD3) [17]	III	≥18 years	Baricitinib (long-term extension of BREEZE-AD1 and BREEZE-AD2)	Patients with IGA 0/1 at week 68: - 2 mg: 59.3% - 4 mg: 47.1%
NCT03428100 (BREEZE-AD4) [18]	III	≥18 years	Baricitinib, placebo, TCS	Patients with EASI-75 at week 16: - 2 mg: 28% - 4 mg: 32% - placebo: 17%
NCT03435081 (BREEZE-AD5) [19]	III	≥18 years	Baricitinib, placebo	Patients with EASI-75 at week 16: - 1 mg: 13% - 2 mg: 30% - placebo: 8%
NCT03559270 (BREEZE-AD6) *	III	≥18 years	Baricitinib	Active, not recruiting
NCT03733301 (BREEZE-AD7) [20]	III	≥18 years	Baricitinib, placebo, TCS	Patients with IGA 0/1 at week 16: - 2 mg: 24% - 4 mg: 31% - placebo: 15%
NCT03952559 (BREEZE-AD Peds) *	III	2–17 years	Baricitinib, placebo, TCS	Recruiting
Upadacitinib (systemic selective JAK 1 inhibitor)				
NCT02925117 [21]	II	18–75 years	Upadacitinib, placebo	EASI improvement at week 16: - 7.5 mg: 39% - 15 mg: 62% - 30 mg: 74% - placebo: 23%
NCT03569293 (Measure Up-1) [22,23]	III	12–75 years	Upadacitinib, placebo	Patients with EASI-75 at week 16: - 15 mg: 70% - 30 mg: 80% - placebo: 16%
NCT03607422 (Measure Up-2) [22,23]	III	12–75 years	Upadacitinib, placebo	Patients with EASI-75 at week 16: - 15 mg: 60% - 30 mg: 73% - placebo: 13%
NCT03568318 (AD Up) [24,25]	III	12–75 years	Upadacitinib, placebo, TCS	Patients with EASI-75 at week 16: - 15 mg: 65% - 30 mg: 77% - placebo: 23% Patients with IGA 0/1 at week 16: - 15 mg: 40% - 30 mg: 59% - placebo: 11%
NCT03738397 (Heads Up) [26]	III	12–75 years	Upadacitinib, dupilumab	Patients with EASI-75 at week 16: - Upadacitinib 30 mg: 71% - Dupilumab: 61.1%
NCT03661138 (Rising Up) [27]	III	12–75 years	Upadacitinib, placebo, TCS	n/a (safety analysis)
NCT04195698 *	III	18–75 years	Upadacitinib	Active, not recruiting
Abrocitinib (systemic JAK 1 inhibitor)				
NCT02780167 [28]	II	18–75 years	Abrocitinib, placebo	Patients with IGA 0/1 at week 12: - 10 mg: 40% (10.9%) - 30 mg: 59% (8.9%) - 100 mg: 29.6% - 200 mg: 43.8% - placebo: 5.8%
NCT04345367 *	III	≥18 years	Abrocitinib, dupilumab	not yet available
NCT03349060 (JADE MONO-1) [29]	III	≥12 years	Abrocitinib, placebo	Patients with IGA 0/1 at week 12: - 100 mg: 44% - 200 mg: 24% - placebo: 8%
NCT03575871 (JADE MONO-2) [30]	III	≥12 years	Abrocitinib, placebo	Patients with IGA 0/1 at week 12: - 100 mg: 44% - 200 mg: 24% - placebo: 8%
NCT03796676 (JADE TEEN) [31]	III	12–17 years	Abrocitinib, placebo	Patients with EASI-75 at week 12: - 100 mg: 44.5% - 200 mg: 61% - placebo: 10.4% Patients with IGA 0/1 at week 12: - 100 mg: 38.1% - 200 mg: 28.4% - placebo: 9.1%
NCT03627767 (JADE REGIMEN) [32]	III	≥12 years	Abrocitinib, placebo	n/a (assessment of treatment modification on AD symptoms/flares)
NCT03720470 (JADE COMPARE) [33]	III	≥18 years	Abrocitinib, dupilumab, placebo	Patients with EASI-75 at week 12: - 100 mg: 58.7% - 200 mg: 70.3% - dupilumab: 58.1% - placebo: 27.1% Patients with IGA 0/1 at week 12: - 100 mg: 36.6% - 200 mg: 48.4% - dupilumab: 36.5% - placebo: 14%
NCT03422822 (JADE EXTEND) [34]	III	≥12 years	Abrocitinib (extension study after switching from dupilumab)	Patients (Dupilumab-responders) with EASI-75 at week 12: - 100 mg: 90.2% - 200 mg: 93.5% Patients (Dupilumab-non-responders) with EASI-75 at week 12: - 100 mg: 67.7% - 200 mg: 80%
NCT05375929 *	III	≥12 years	Abrocitinib	Not yet recruiting
SHR0302 (systemic selective JAK 1 inhibitor)				
NCT04717310 (MARBLE-23) *	II/III	≥12	SHR0302 (oint), vehicle	Recruiting
NCT04162899 [35]	II	18–75	SHR0302	Patients with IGA 0/1 at week 12: - 4 mg: 25.7% - 8 mg: 54.3% - placebo: 5.7%
NCT04875169 *	III	≥12	SHR0302, placebo	Active, not recruiting

EASI: Eczema Area and Severity Index; IGA: Investigator’s Global Assessment; mEASI: modified Eczema Area and Severity Index; n/a: not applicable; TCS: topical corticosteroids; * Additional, unpublished RCTs listed on https://www.clinicaltrials.gov, accessed on 10 June 2022.

**Table 3:** JAK selectivity of systemic JAK inhibitors.

JAK Inhibitor	IC_50_ Values (nM)				Indications
	JAK1	JAK2	JAK3	TYK2	
Baricitinib (JAK1/2)	4.0	6.6	787	61	AD
Upadacitinib (JAK1)	43	120	2300	4700	AD, RA, AS
Abrocitinib (JAK1)	29	803	>10.000	1259	AD
Tofacitinib (JAK1/2/3)	15	77	55	489	RA, AS, PsA, UC, JIA

AD: atopic dermatitis, AS: ankylosing spondylitis, JAK: janus kinase, JIA: juvenile idiopathic arthritis, PsA: psoriatic arthritis, RA: rheumatoid arthritis, TYK2: tyrosine kinase 2, UC: ulcerative colitis.

## Abbreviations

AD	atopic dermatitis
BSA	body surface area
CPK	creatine phosphokinase
EASI	eczema area and severity index
IFN-γ	interferon-γ
Ig	Immunoglobulin
IGA	Investigator’s Global Assessment
IL	Interleukin
JAK	Janus kinase
RCT	randomized controlled trial
STAT	signal transducer and activation of transcription
TCS	topical corticosteroids
Th	T helper
TNF-a	tumor necrosis factor alpha
Treg	T regulatory
TSLP	thymic stromal lymphopoietin
TYK2	tyrosine kinase 2
